# Outcomes of functional ventricular tachycardia ablation vs. medical therapy in Chagas cardiomyopathy patients with implantable cardioverter-defibrillators: a competing risks analysis

**DOI:** 10.1093/europace/euaf311

**Published:** 2025-12-02

**Authors:** Gustavo de Araújo Silva, Bruno Wilnes, Beatriz Castello-Branco, José Luiz Padilha da Silva, Marina Pereira Mayrink, Anna Terra França, Marcos Roberto Queiroz França, Isabella Moreira Gonzalez Fonseca, Reynaldo Castro de Miranda, Maria do Carmo Pereira Nunes, Andre Assis Lopes Carmo

**Affiliations:** Post Graduate Program in Infectious Diseases and Tropical Medicine, School of Medicine, Universidade Federal de Minas Gerais, Av. Professor Alfredo Balena, 190, Belo Horizonte, MG 30130-100, Brazil; Unit of Cardiology and Cardiovascular Surgery, Hospital das Clinicas, Universidade Federal de Minas Gerais, Av. Prof. Alfredo Balena, 110, Belo Horizonte, MG 30130-100, Brazil; Advanced Arrhythmia Treatment Center (CTA), Av. Barbacena, 472, Belo Horizonte, MG 30190-130, Brazil; Advanced Arrhythmia Treatment Center (CTA), Av. Barbacena, 472, Belo Horizonte, MG 30190-130, Brazil; Advanced Arrhythmia Treatment Center (CTA), Av. Barbacena, 472, Belo Horizonte, MG 30190-130, Brazil; Department of Statistics, Universidade Federal do Paraná, R. Evaristo F. Ferreira da Costa, 408, Curitiba, PR 81530-015, Brazil; Advanced Arrhythmia Treatment Center (CTA), Av. Barbacena, 472, Belo Horizonte, MG 30190-130, Brazil; Unit of Cardiology and Cardiovascular Surgery, Hospital das Clinicas, Universidade Federal de Minas Gerais, Av. Prof. Alfredo Balena, 110, Belo Horizonte, MG 30130-100, Brazil; Advanced Arrhythmia Treatment Center (CTA), Av. Barbacena, 472, Belo Horizonte, MG 30190-130, Brazil; Unit of Cardiology and Cardiovascular Surgery, Hospital das Clinicas, Universidade Federal de Minas Gerais, Av. Prof. Alfredo Balena, 110, Belo Horizonte, MG 30130-100, Brazil; Advanced Arrhythmia Treatment Center (CTA), Av. Barbacena, 472, Belo Horizonte, MG 30190-130, Brazil; Post Graduate Program in Infectious Diseases and Tropical Medicine, School of Medicine, Universidade Federal de Minas Gerais, Av. Professor Alfredo Balena, 190, Belo Horizonte, MG 30130-100, Brazil; Advanced Arrhythmia Treatment Center (CTA), Av. Barbacena, 472, Belo Horizonte, MG 30190-130, Brazil; Advanced Arrhythmia Treatment Center (CTA), Av. Barbacena, 472, Belo Horizonte, MG 30190-130, Brazil; Post Graduate Program in Infectious Diseases and Tropical Medicine, School of Medicine, Universidade Federal de Minas Gerais, Av. Professor Alfredo Balena, 190, Belo Horizonte, MG 30130-100, Brazil; Unit of Cardiology and Cardiovascular Surgery, Hospital das Clinicas, Universidade Federal de Minas Gerais, Av. Prof. Alfredo Balena, 110, Belo Horizonte, MG 30130-100, Brazil; Post Graduate Program in Infectious Diseases and Tropical Medicine, School of Medicine, Universidade Federal de Minas Gerais, Av. Professor Alfredo Balena, 190, Belo Horizonte, MG 30130-100, Brazil; Unit of Cardiology and Cardiovascular Surgery, Hospital das Clinicas, Universidade Federal de Minas Gerais, Av. Prof. Alfredo Balena, 110, Belo Horizonte, MG 30130-100, Brazil; Advanced Arrhythmia Treatment Center (CTA), Av. Barbacena, 472, Belo Horizonte, MG 30190-130, Brazil

**Keywords:** Chagas cardiomyopathy, Ventricular tachycardia ablation, Functional mapping

## Introduction

Chagas cardiomyopathy (ChC), a fibrotic–inflammatory condition that follows *Trypanosoma cruzi* infection, carries a high arrhythmic burden, with sustained ventricular tachycardia (VT) as a principal driver of sudden death.^1,2^ Implantable cardioverter-defibrillators (ICDs) are often used for secondary prevention, but frequent VT recurrences and ICD shocks may exacerbate ventricular dysfunction and adversely affect survival.^3,4^ In this context, catheter ablation has emerged as an alternative to reduce arrhythmic burden, and recent advances in mapping techniques have led to functional substrate mapping, which integrates both static and dynamic electrophysiological properties.^5,6,7^ This approach extends beyond voltage-defined scar zones by targeting abnormal electrograms, with the potential to improve ablation outcomes.

Pharmacological therapy, most often with amiodarone, remains widely used; however, its effect on mortality is inconsistent.^8^ Moreover, ICD therapy itself has not been shown to reduce all-cause mortality in ChC, underscoring the need for interventions that minimize VT recurrence and ICD therapies.^9,10^

Against this background, the present study sought to (i) compare VT recurrence and mortality in ChC patients undergoing ablation vs. medical therapy, (ii) assess outcomes of conventional vs. functional VT ablation, and (iii) evaluate the prognostic impact of early VT recurrence post-ablation. A competing risks approach was used to account for mortality.

## Methods

Adult patients (age ≥ 18) with ChC and sustained ventricular arrhythmias requiring ICDs for secondary prevention were enrolled between 2017 and 2022. Diagnosis was based on positive serology and typical cardiomyopathy criteria. Exclusion criteria included overlapping aetiologies. Of 254 screened patients, 127 met inclusion criteria and were divided into the ablation group (cases) and medical treatment group (controls). The ablation group was further stratified into the voltage-based approach [45 patients; 43 (95.5%) with epicardial mapping] and functional mapping approach [22 patients; 19 (86.4%) with epicardial mapping].

Follow-up was for up to 24 months. Ventricular tachycardia recurrence was defined as any sustained VT treated by ICD or documented below device detection threshold. The primary outcome was VT recurrence; death was treated as a competing risk. Secondary outcomes are all-cause mortality and the composite of VT recurrence and all-cause mortality.

The functional map was constructed using strategies to assess wavefront conduction patterns and velocity through the myocardium, incorporating programmed extra-stimulation and systematically identifying regions of late potentials (LPs), decremental conduction, and deceleration zones. In addition, these electrophysiological properties were analysed during the patient’s intrinsic rhythm. Voltage-based mapping defined scars via bipolar and unipolar thresholds.

The voltage-based strategy group consisted of patients previously treated with scar homogenization for VT ablation. Substrate mapping during intrinsic rhythm was performed to delineate ventricular scar, with fractionated potentials and LPs annotated to guide lesion delivery. Procedural success was assessed by the elimination or substantial modification of abnormal electrograms and by programmed electrical stimulation (PES). Patients included in the medical therapy group were those who, after ICD implantation, experienced at least one episode of ventricular arrhythmia as previously described and were subsequently maintained on amiodarone therapy.

Statistical analyses used Fine–Gray models for competing risks and Cox regression for mortality predictors. Adjustments included PAINESD (Pulmonary disease, Age >60 years, Ischemic cardiomyopathy, NYHA III–IV, Ejection fraction ≤25%, electrical Storm, Diabetes) score components, which were incorporated into the multivariable analysis. Cumulative incidence function curves and Kaplan–Meier survival analyses were applied.

## Results

The ablation group had more advanced heart failure [37% New York Heart Association (NYHA) III/IV] and higher incidence of electrical storms (87% vs. 17%), as shown in *Table 1*. The overall mean PAINESD score was 10 ± 4.6. The PAINESD score was not significantly different between voltage-based and functional mapping groups (10.7 ± 4.6 vs. 8.6 ± 4.4; *P* = 0.074). Mechanical circulatory support with intra-aortic balloon pump was required in five patients (7.5%) overall—three (6.7%) in the voltage-based group and two (9.1%) in the functional mapping group—with no difference between groups (*P* = 1.0). Ventricular tachycardia recurrence occurred in 69 patients (54%), with 83 experiencing the composite outcome. There were 40 deaths, with 26 following VT recurrence.

**Table 1:** Baseline characteristics of the study population stratified by treatment group

Variables	Catheter ablation (*n* = 67)	Medical therapy (*n* = 60)	*P* value
Age (years)	63.0 (55.0–72.0)	63.5 (54.3–73.0)	0.987
Male sex	34 (50.7)	44 (73.3)	0.009
NYHA class III/IV	25 (37.3)	9 (15.0)	0.005
LV ejection fraction (%)	37.0 (30.0–46.0)	39.0 (32.0–49.0)	0.214
Comorbidities			
Hypertension	16 (23.9)	22 (36.7)	0.116
Diabetes	4 (6.0)	7 (11.7)	0.347
eGFR < 60 mL/min	16 (23.9)	10 (16.7)	0.314
Atrial fibrillation	13 (19.4)	16 (26.7)	0.330
Thyroidopathy	23 (34.3)	19 (31.7)	0.750
COPD	4 (6.0)	4 (6.7)	1.000
Stroke	7 (10.4)	6 (10.0)	1.000
Medications			
Beta-blockers	65 (97.0)	57 (95.0)	0.667
ACEI or ARB	51 (76.1)	46 (76.7)	0.942
Propafenone	1 (1.5)	0 (0)	1.000
Spironolactone	34 (50.7)	26 (43.3)	0.403
Anticoagulation	7 (10.4)	24 (40.0)	<0.001
Diuretic	32 (47.8)	33 (55.0)	0.415
Amiodarone use	60 (89.6)	49 (83.1)	0.286
Amiodarone dose (mg)	400 (400–800)	200 (100–200)	<0.001
Previous ventricular arrhythmia			
Incessant VT	11 (16.4)	1 (1.8)	0.006
VT below the detection zone	21 (31.3)	9 (15.3)	0.034
VT storm	58 (86.6)	10 (16.7)	<0.001

ACEI, angiotensin-converting enzyme inhibitor; ARB, angiotensin II receptor blocker; COPD, chronic obstructive pulmonary disease; eGFR, estimated glomerular filtration rate; LV, left ventricular; NYHA, New York Heart Association; VT, ventricular tachycardia.

**Figure 1:**
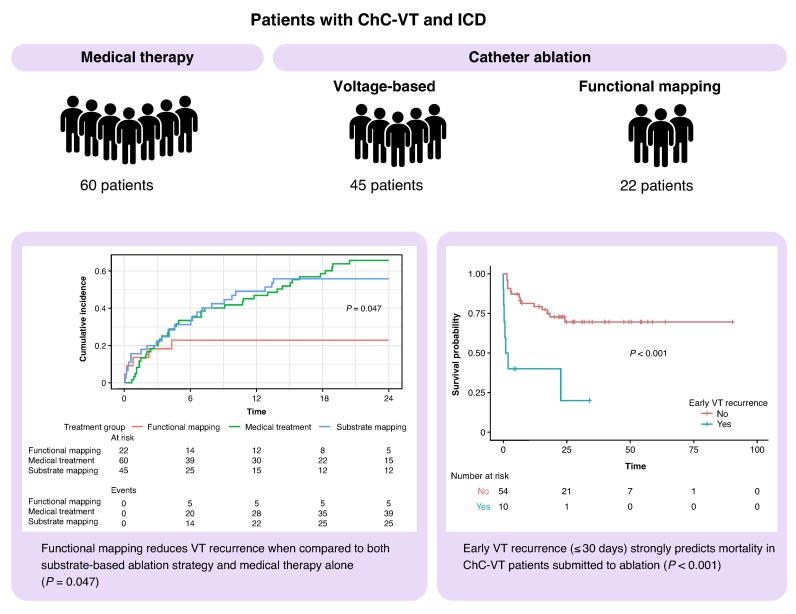
Study population and key outcomes in patients with ChC-VT and ICD. The cohort comprised 127 patients divided into three groups: medical therapy (*n* = 60), voltage-based substrate ablation (*n* = 45), and functional mapping-guided ablation (*n* = 22). Left panel: Cumulative incidence of VT recurrence according to treatment strategy. Functional mapping significantly reduced VT recurrence compared with both voltage-based ablation and medical therapy alone (*P* = 0.047). Right panel: Kaplan–Meier survival curve showing that early VT recurrence (≤30 days) after ablation strongly predicted mortality in ChC-VT patients (*P* < 0.001). ChC-VT, Chagas cardiomyopathy-related ventricular tachycardia; ICD, implantable cardioverter-defibrillator; VT, ventricular tachycardia.

Ventricular tachycardia recurrence at 24 months was lower with functional ablation (HR 0.332, 95% CI 0.121–0.901, *P* = 0.047), resulting in a 76.2% arrhythmia-free survival vs. 32.3% in the medical group (*Figure 1*). Functional ablation was also associated with a reduced composite outcome of VT recurrence or death (68.2% event-free vs. 28%). Multivariable analysis confirmed functional ablation as an independent predictor of lower VT recurrence (HR 0.236, 95% CI 0.079–0.709, *P* = 0.010).

Mortality was similar between groups overall. However, VT recurrence within 30 days post-ablation was independently associated with higher mortality (HR 3.247, 95% CI 1.216–8.665, *P* < 0.001), as shown in *Figure 1*.

## Discussion

Patients with ChC receiving ICDs have high rates of VT recurrence. Functional ablation, targeting dynamically abnormal regions beyond voltage-defined scar, was associated with significantly lower VT recurrence.

Patients with ChC and ICDs continue to experience high VT recurrence when treated with medical therapy alone. In our cohort, arrhythmia-free survival at 24 months was only 32% with drug therapy, underscoring its limited efficacy. Ablation strategies provided greater arrhythmia control, but the benefit was driven predominantly by functional mapping. Patients treated with functional ablation achieved markedly better outcomes, with arrhythmia-free survival exceeding 76%, significantly outperforming both medical therapy and conventional ablation. This findings support the notion that targeting dynamically abnormal electrograms beyond static scar borders is important to optimize substrate modification in ChC.

Although overall mortality did not differ between ablation and medical therapy, patients who underwent ablation had a significantly more severe clinical profile, including more advanced heart failure and a higher incidence of electrical storms. Early VT recurrence within 30 days of ablation was independently associated with increased risk of death, emphasizing the prognostic importance of effective substrate modification. Taken together, these findings suggest that functional ablation is associated with superior arrhythmia control in ChC, while highlighting the need for timely intervention and close monitoring of patients with early recurrence.

## Conclusions

In ChC, medical therapy alone was associated with high rates of VT recurrence. Functional VT ablation is associated with a significantly reduced VT recurrence and outperformed both medical therapy and conventional ablation. Early post-ablation recurrence identified patients at highest risk of adverse outcomes, underscoring the importance of durable arrhythmia control.

## Data Availability

The datasets generated and/or analysed during the current study will be made available from the corresponding author upon reasonable request.

## References

[1] Nunes MCP, Beaton A, Acquatella H, Bern C, Bolger AF, Echeverría LE et al Chagas cardiomyopathy: an update of current clinical knowledge and management: a scientific statement from the American Heart Association. Circulation 2018;138:e169–209. 30354432 10.1161/CIR.0000000000000599

[2] Könemann H, Dagres N, Merino JL, Sticherling C, Zeppenfeld K, Tfelt-Hansen J et al Spotlight on the 2022 ESC guideline management of ventricular arrhythmias and prevention of sudden cardiac death: 10 novel key aspects. Europace 2023;25:euad091. 37102266 10.1093/europace/euad091 PMC10228619

[3] Barbosa MPT, Carmo AAL, Rocha MOC, Ribeiro ALP. Ventricular arrhythmias in Chagas disease. Rev Soc Bras Med Trop 2015;48:4–10. 10.1590/0037-8682-0003-2014 25714933

[4] Carmo AAL, de Sousa MR, Agudelo JF, Boersma E, Rocha MOC, Ribeiro ALP et al Implantable cardioverter-defibrillator in Chagas heart disease: a systematic review and meta-analysis of observational studies. Int J Cardiol 2018;267:88–93. 29871807 10.1016/j.ijcard.2018.05.091

[5] Wilnes B, Castello-Branco B, Pereira EMM, Lopes LM, Santos VB, Bicalho AC et al Extrastimuli-assisted functional mapping improves ventricular tachycardia ablation outcomes: a systematic review, meta-analysis, and meta-regression. Heart Rhythm 2025. 10.1016/j.hrthm.2025.03.2000 40188999

[6] Wilnes B, Castello-Branco B, Silva GA, Mayrink M, Silva JLP, Barbosa MPT et al Enhancing ventricular tachycardia ablation outcomes. JACC Clin Electrophysiol 2025;11:200–2. 39614864 10.1016/j.jacep.2024.09.030

[7] Srinivasan NT, Garcia J, Schilling RJ, Ahsan S, Babu GG, Ang R et al Multicenter study of dynamic high-density functional substrate mapping improves identification of substrate targets for ischemic ventricular tachycardia ablation. JACC Clin Electrophysiol 2020;6:1783–93. 33357574 10.1016/j.jacep.2020.06.037 PMC7769061

[8] Wiedmann F, Ince H, Stellbrink C, Kleemann T, Eckardt L, Brachmann J et al Single beta-blocker or combined amiodarone therapy in implantable cardioverter-defibrillator and cardiac resynchronization therapy—defibrillator patients: insights from the German DEVICE registry. Heart Rhythm 2023;20:501–9. 36509321 10.1016/j.hrthm.2022.12.009

[9] França AT, Martins LNA, de Oliveira DM, de Castilho FM, Branco BC, Wilnes B et al Evaluation of patients with implantable cardioverter-defibrillator in a Latin American tertiary center. J Cardiovasc Electrophysiol 2024;35:675–84. 38323491 10.1111/jce.16201

[10] Martinelli-Filho M, Marin-Neto JA, Scanavacca MI, de Paola AAV, Medeiros P, Owen R et al Amiodarone or implantable cardioverter-defibrillator in Chagas cardiomyopathy. JAMA Cardiol 2024;9:1073. 39356542 10.1001/jamacardio.2024.3169 PMC11447631

